# Does urethral length affect continence outcomes following robot assisted laparoscopic radical prostatectomy (RALP)?

**DOI:** 10.1186/s12894-020-0578-x

**Published:** 2020-01-31

**Authors:** Diwei Lin, Michael O’Callaghan, Rowan David, Andrew Fuller, Richard Wells, Peter Sutherland, Darren Foreman

**Affiliations:** 1 Flinders Medical Centre, Adelaide, South Australia Australia; 2 South Australia Prostate Cancer Clinical Outcomes Collaborative, Flinders Medical Centre, Adelaide, South Australia Australia; 3 Flinders Centre for Innovation in Cancer, Adelaide, South Australia Australia; 4 University of Adelaide, Discipline of Medicine, Adelaide, South Australia Australia; 5 South Terrace Urology, Adelaide, South Australia Australia

**Keywords:** Incontinence, Post-prostatectomy, Robotic-prostatectomy, Urethral length, Urology

## Abstract

**Background:**

Post-operative urinary incontinence is a significant concern for patients choosing to undergo a radical prostatectomy (RP) for treatment of prostate cancer. The aim of our study was to determine the effect of pre-operative MUL on 12 month continence outcomes in men having robot-assisted laparoscopic prostatectomy (RALP).

**Methods:**

We use the South Australian Prostate Cancer Clinical Outcomes Collaborative (SA-PCCOC) database, to identify 602 patients who had undergone RALP by a high volume surgeon. Only patients who received an assessment and education by a specialist pelvic floor physiotherapist, had completed EPIC questionnaires before treatment and did not have radiotherapy treatment within 12 months of surgery were included. MUL measurements were taken from pre-operative magnetic resonance imaging (MRI) scans. The short-form version of the Expanded Prostate Cancer Index Composite (EPIC-26) was used to measure continence outcomes. Continence was defined as 100/100 in the EPIC-26 Urinary Continence domain score.

**Results:**

The observed median MUL in this study was 14.6 mm. There was no association between MUL and baseline continence. MUL was associated with continence at 12 months post RALP (OR 1.13, 95% CI 1.03–1.21, *p* = 0.0098). In men who were continent before surgery, MUL was associated with return to continence at 12 months after RALP (OR 1.15, 1.05–1.28, *p* = 0.006). MUL was also associated with change in continence after surgery (β = 1.22, *p* = 0.002).

**Conclusions:**

MUL had no effect on baseline continence but had a positive and significant association with continence outcomes over 12 months post RALP.

## Background

Post-operative urinary incontinence is a significant issue for patients choosing to undergo a radical prostatectomy for treatment of prostate cancer. Many factors such as age, body mass index (BMI) and prostate volume are thought to affect continence outcomes following radical prostatectomy [1]. Membranous urethral length (MUL) measured prior to radical prostatectomy has recently been identified as an additional factor by numerous authors [2]. A recent systematic review and meta-analysis of all studies reporting the effect of MUL on the recovery of continence following RP found a greater preoperative MUL is significantly and positively associated with an earlier return to continence [2]. The aim of our study was to determine the effect of pre-operative MUL on urinary continence at12 months after RALP.

## Methods

### Patients

The South Australian Prostate Cancer Clinical Outcomes Collaborative (SA-PCCOC) prospectively collects clinical and oncological information on RALP cases performed in both the public and private sector in South Australia, Australia. Utilising this database, we identified patients who fulfilled the following inclusion criteria: they had undergone RALP by a high volume surgeon (> 50 cases per annum), received an assessment and education by a specialist pelvic floor physiotherapist, had completed EPIC questionnaires before treatment and did not have radiotherapy treatment within 12 months of surgery.

### MUL measurements

Accurate MUL measurements were taken from the patients’ pre-operative magnetic resonance imaging (MRI) scans. To maintain consistency with the published literature, measurements were taken in an identical fashion to other authors. The MUL was measured along a straight line between the prostatic apex and the penile bulb in both the mid-sagittal plane and the coronal plane. A consultant radiologist demonstrated how measurements should be taken and the readings were recorded separately by two independent reviewers (DL and RD) and cross-checked. Any discrepancy of more than 10% resulted in a repeat measurement to obtain a third reading.

### Patient reported outcome measures

The 26-item short-form version of the Expanded Prostate Cancer Index Composite (EPIC-26) was used to measure outcomes. The EPIC is a validated tool used to assess quality of life outcomes in men who have undergone treatment for prostate cancer [3]. The four questions listed below from EPIC-26 pertain to urinary continence:

- Q23: Over the past 4 weeks how often have you leaked urine?

More than once a day (1); Once a day (2); More than once a week (3); About once a week (4); Rarely or never (5)

- Q26: Which of the following best describes your control during the last 4 weeks?

None (1); Frequent dribbling (2); Occasional dribbling (3); Total control (4)

- Q27: How many pads or diapers per day did you use during the last 4 weeks?

None (0); 1 per day (1); 2 per day (2); 3 or more (3)

- Q28: How big a problem during the last 4 weeks had dripping or leaking of urine been?

No problem (0); Very small problem (1); Small problem (2); Moderate problem (3); Big problem (4)

The highest mark for each question was given 100 points. The total score was calculated as an average of each question. Continence was defined as 100/100 in the EPIC26 Urinary Continence domain score. The EPIC26 was repeated at baseline, 3, 6 and 12 months. Baseline was defined as any time after the date of diagnosis and prior to RALP.

### Statistics

Linear regression was used to assess correlation between sagittal and coronal measurements of MUL. To assess the association of MUL with continence at baseline and separately at 12 months post RALP, a logistic regression model was used. Continence was deemed to be and EPIC-26 score of 100 before treatment, and incontinence a score less than 100. A linear model was used to assess change in continence between baseline and 12 months post treatment. To assess continence from 3 to 12 months a mixed effect linear model was used. A further logistic regression model was used to assess return to baseline continence in the subset of men who were continent prior to surgery. Statistical analysis was performed in R and *p* < 0.05 considered to be statistically significant.

### Ethics

This study uses data collected retrospectively as part of the South Australian Prostate Cancer Clinical Outcomes Collaborative Database. The database has been reviewed and approved by the Southern Adelaide Human Research Ethics Committee (307.14). The database informs participants about the study, and uses an approved opt-out consent process. Informed consent was obtained from all individual participants included in the study (through the opt-out mechanism). All procedures performed in studies involving human participants were in accordance with the ethical standards of the institutional and/or national research committee (Southern Adelaide Human Research Ethics Committee (307.14)) and with the 1964 Helsinki declaration and its later amendments or comparable ethical standards.

## Results

### Demographics

The effect of MUL on continence outcomes was evaluated in 602 patients. The mean age at diagnosis was 64 years old. 74.5% of patients had clear oncological margins. 72.5% of patients had either a uni- (34%) or bilateral (38%) nerve sparing procedures. The mean prostate volume was 42.5 g. The mean coronal MUL was 14.6 mm. (Table 1).

**Table 1:** Demographics and measurements

		Overall
n		602
Age at diagnosis (mean (sd))		64.03 (6.63)
Margin status (n,%)	Clear	347 (74.5)
	Involved	119 (25.5)
Nerve sparing (n,%)	Bilateral	85 (38.3)
	No	61 (27.5)
	Unilateral	76 (34.2)
Prostate volume (mean (sd))		42.56 (21.15)
Coronal length (mean (sd))		14.62 (3.61)
Sagittal length (mean (sd))		14.64 (3.66)

### Measurements

**Fig. 1:**
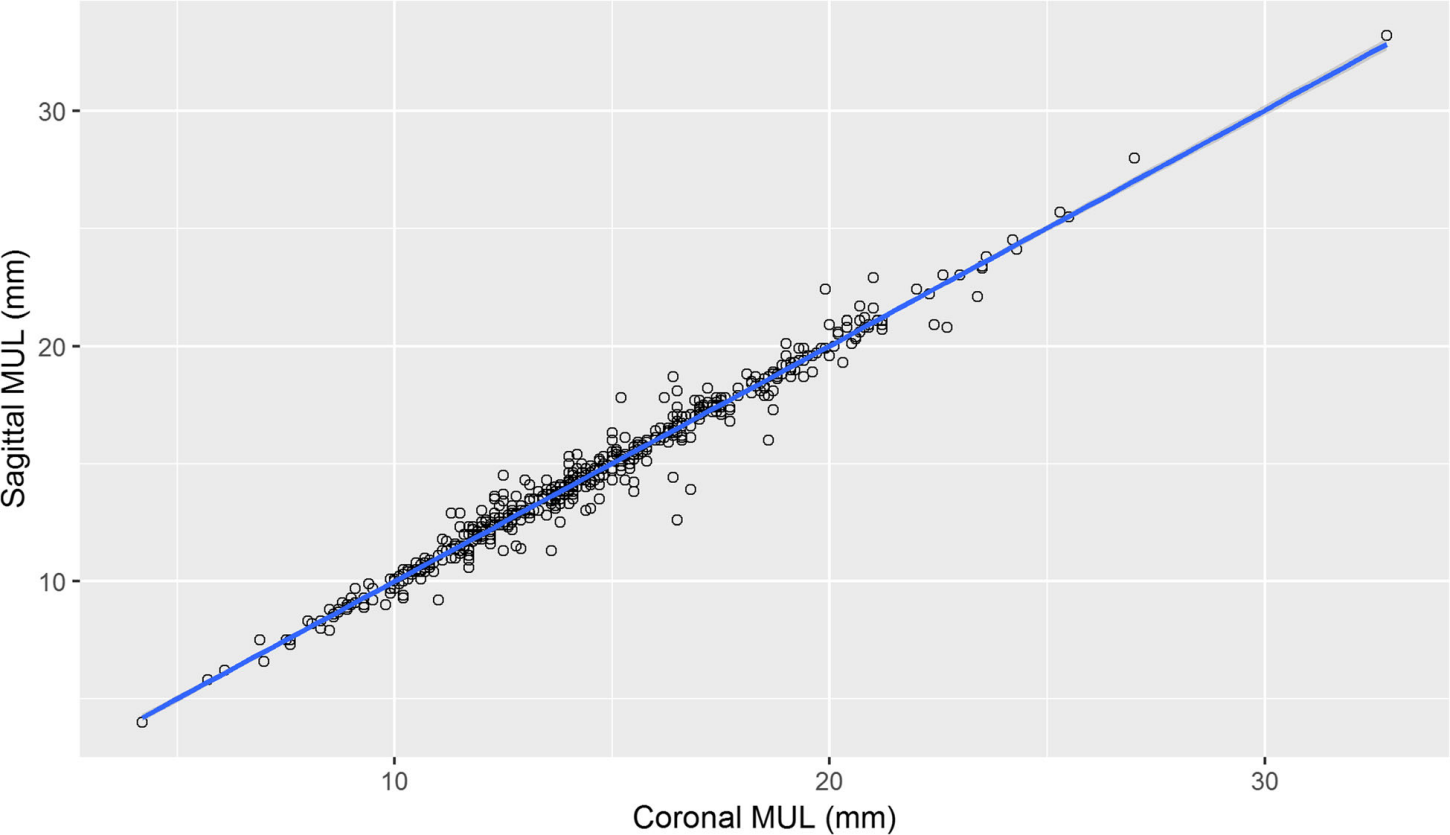
Urethral length measurement correlation. There was a near perfect correlation between the two measurements of the urethral length in the coronal and sagittal planes. *n* = 602. To maintain consistency with the published literature, coronal measurements are used throughout this analysis. β = 0.97

There was a near perfect correlation between the two measurements of the urethral length in the coronal and sagittal planes. (Table 1, Fig. 1) To maintain consistency with the published literature, coronal measurements are used throughout this analysis.

### Effect of urethral length on urinary continence at baseline

**Fig. 2:**
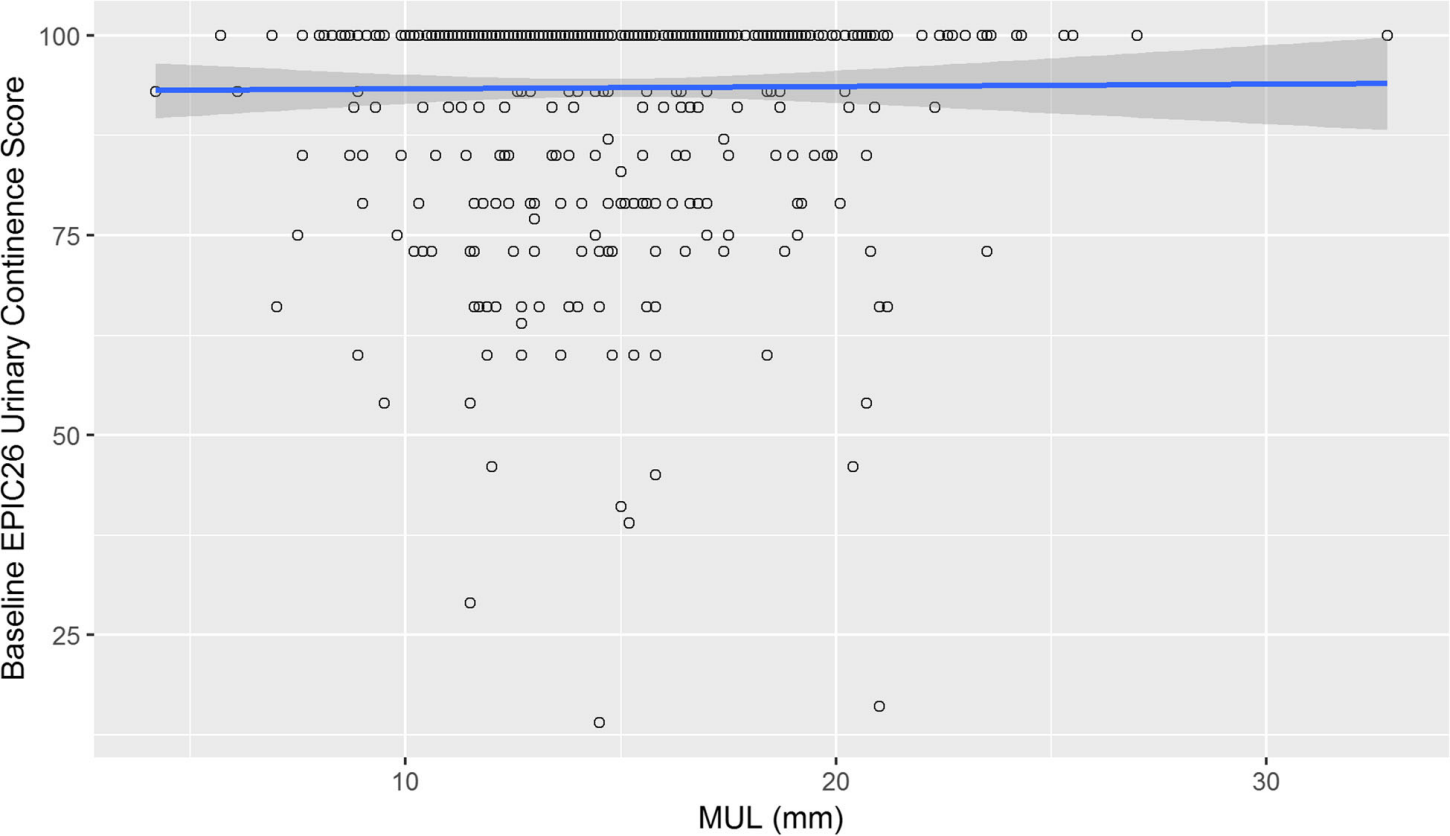
Relationship between MUL and baseline continence. A logistic and linear regression of the sample group showed no significant association between MUL and continence at baseline

Linear regression of the sample group showed no significant association between MUL and continence at baseline (Fig. 2, β = 0.03, *p* = 0.85).

### Continence post RALP over 12 months

**Fig. 3:**
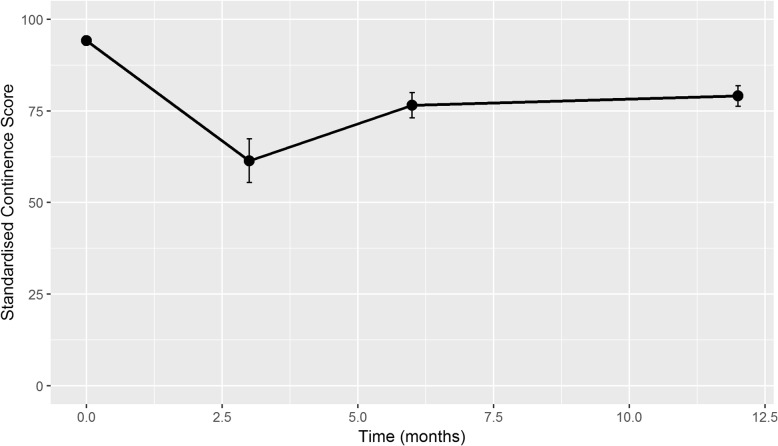
Change in continence scores post RALP. Continence scores dropped off most markedly at 3 months post RALP, mostly recovering by 6 months with minimal improvement from six to 12 months

Continence scores dropped off most markedly at 3 months post RALP, mostly recovering by 6 months with minimal improvement from six to 12 months. (Fig. 3) The average EPIC-26 score at baseline was 94. This dropped to 61 at 3 months before recovering to 76 at 6 months and reaching 79 at 12 months post RALP. Of note, a low number of questionnaires were returned (*n* = 75) at 3 months. (Table 2).

**Table 2:** Post-RALP EPIC-26 continence scores

Time (months)	n	Mean EPIC 26 score	SD
0	365	94	11.83
3	75	61	25.83
6	183	76	23.68
12^a^	242	79	22.02

^a^At 12 months 92/242 men achieved an EPIC26 score of 100/100

### Effect of urethral length on continence post RALP over 12 months

After adjustment for age and time, there was a significant effect of MUL on continence post RP, using a mixed effects linear model and continence measured at 3, 6 and 12 months (β = 0.607, *p* = 0.017,Table 3). This association remained significant after further adjustment for continence measured at baseline (Table 3).

**Table 3:** Continence post RALP over 12 months

	β	*p* value	95% CI
Coronal length (mm)	0.51	0.019	0.08–0.94
Age (years)	0.72	< 0.001	0.59–0.86
Time (months)	−0.22	< 0.001	−1.47- -0.95

### Change in continence at 12 months compared to baseline

**Fig. 4:**
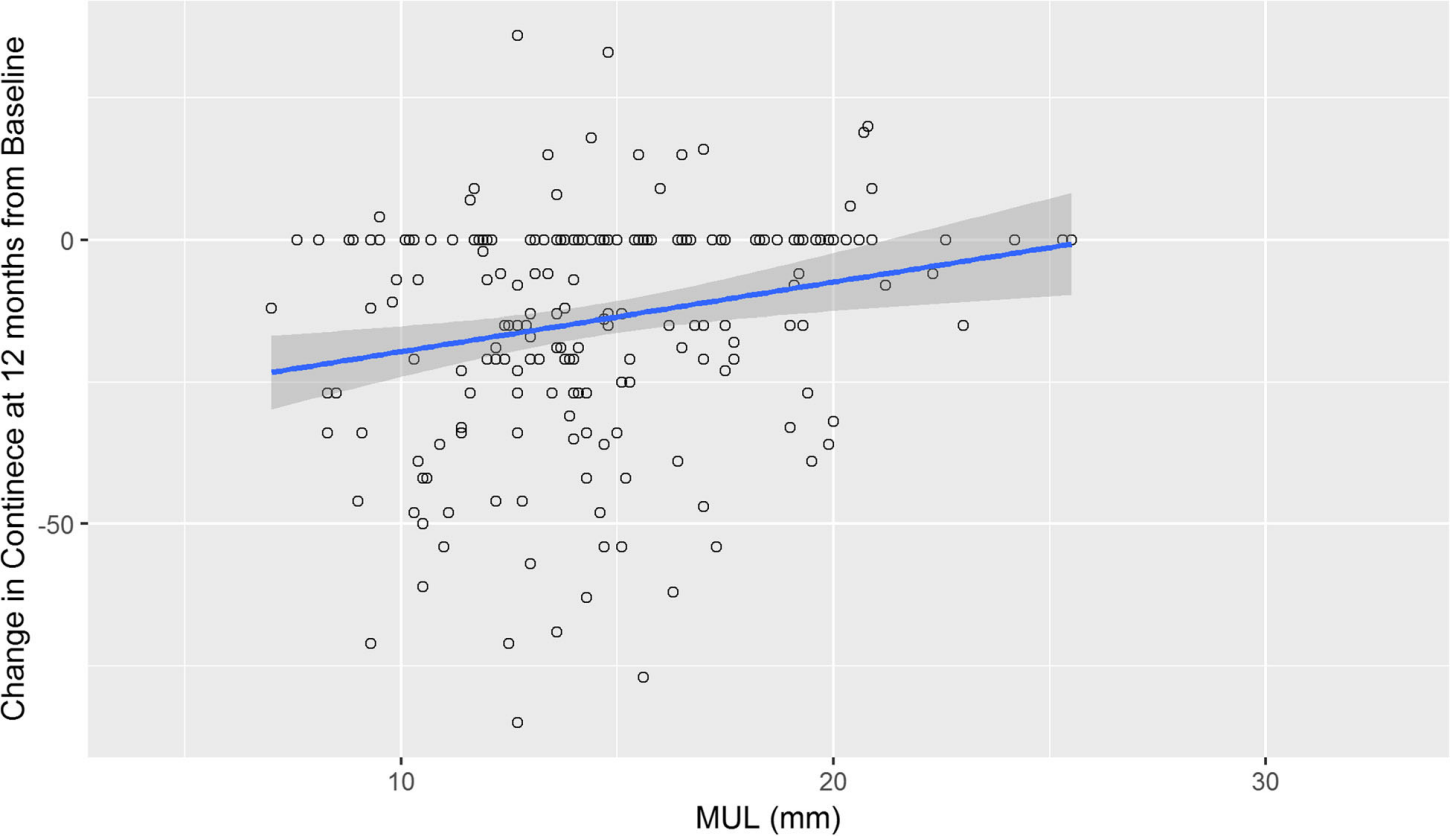
MUL and change in continence scores at 12 months. There was a significant association between MUL and change in EPIC-26 score between baseline and 12 months post RALP in a linear regression model. (β = 0.03, *p* = 0.002)

There was a significant association between MUL and change in EPIC-26 score between baseline and 12 months post RALP in a linear regression model. (β = 0.03, *p* = 0.002, Fig. 4). Being continent at 12 months post RALP was also associated with MUL length (OR 1.11, 95% CI 1.02–1.21, *p* = 0.009, Table 4). This association remained significant with additional adjustment for prostate size and nerve sparing status (OR 1.4 1.13–1.94, p = 0.009). Furthermore, in patients who were continent at baseline, by constructing a logistic regression to predict return to continence at 12 months post RALP, a significant association with urethral length was observed. (OR 1.15, 95% CI 1.045–1.28, *p* = 0.006, Table 5).

**Table 4:** Continence at 12 months post RALP (*n* = 208)

	OR	*p* value	95% CI
Coronal length (mm)	1.22	0.011	1.06–1.46
Age (years)	1.02	0.6	0.95–1.10
Baseline continence	1.08	0.015	1.02–1.16

**Table 5:** Recovery of continence at 12 months in those continent at baseline (*n* = 147)

	OR	*p* value	95% CI
Coronal length (mm)	1.25	0.013	1.07–1.53
Age (years)	0.99	0.86	0.92–1.08

## Discussion

The results of this study suggest MUL does not affect continence at baseline. There is however a significant association of MUL with continence after RALP. Furthermore, in men who were continent before surgery, MUL was significantly associated with a return to continence at 12 months post-surgery (OR 1.25, *p* = 0.013).

A literature review of the current evidence supports a positive relationship between a longer MUL and improved continence post RALP. Mungovan et al. have published a systematic review comparing MUL with return of continence [2]. Four studies (*n* = 1738) which reported hazard ratio results found every extra mm of MUL was associated with a faster return to continence (hazard ratio: 1.05; *p* < 0.001). Eleven studies (*n* = 6993) reported the odds ratio (OR) for return to continence at a set time point post RALP: 3 months (OR: 1.08, *p* = 0.004), 6 months (OR: 1.12, *p* < 0.0001), 12 months (OR: 1.12, *p* = 0.006). All OR data combined indicated that every extra mm of MUL was associated with significantly greater odds for return to continence (OR: 1.09, *p* < 0.001). Coakley et al. published a single surgeon case series (*n* = 211) showing that MUL was related to time to stable postoperative continence, graded on a scale of 1 to 5 (*p* = 0.02) [4]. These results are consistent with the findings from our study and suggest that there may now be sufficient evidence to build clinical models predicting urinary continence post-surgery. This endeavour will require a large data set with adequate external validation in a second cohort, and will likely need a collaborative approach involving multiple centres to achieve sufficient statistical power.

Many patient, clinical and disease factors have been identified as negative predictors of post RALP continence outcomes. These include advancing age, previous urethral stricture disease, obesity, low surgeon volume, previous prostate surgery and surgical margin involvement [1, 5, 6]. In addition, some variables may predict continence, but are not known until after surgery (e.g. margin status), limiting their usefulness in pre-operative counselling. To improvement post RALP outcomes, many surgical modifications have been described. Techniques including “reconstructing” the pelvic floor, bladder neck preservation, bladder neck intussusception, bladder neck mucosal eversion, posterior and anterior fixation of the urethra have been utilised with varying success [7, 8]. An additional area for future investigation is the interaction of MUL with nerve sparing status. This may provide important predictive information, though we did not have a sufficient sample size to investigate this in detail in this cohort. If such a cohort is developed, collecting data relating to anatomy of the apex, previous TURP treatment, and use of ROCCO stich may be important to collect as additional potential confounders.

The exact point where the urethra should be divided in order to maximise residual urethral length without compromising surgical margins has not been well-described in the literature. Sfoungaristos et al. describe the verumontanum as their proximal limit for dissection as it is easily recognised, without anatomical variation and represents the anatomical landmark of the striated sphincter [9].

## Conclusion

This is one of the largest studies investigating continence outcomes in patients undergoing RALP and its association with MUL. Strict inclusion criteria and definitions of continence provide the basis for an accurate analysis of post-operative continence outcomes. Membranous urethral length had no effect on baseline urinary incontinence but had a positive and significant association with continence outcomes over 12 months post RALP. The findings of this study will aid in pre-RALP assessment of patients, particularly where clinicians can employ an evidence-based approach to counselling patients regarding incontinence and recovery times based on MUL measurements. By educating patients who might take longer to regain continence based on their MUL measurements, this could potentially improve their psychological well-being and subsequently their overall quality of life. Future studies measuring ‘bother’ scores of the EPIC questionnaire could test the efficacy of such counselling.

## Data Availability

Data that forms part of this manuscript is available upon request, subject to data release conditions.

## Abbreviations

BMI: Body Mass Index
EPIC: Expanded Prostate Cancer Index Composite
MUL: Membranous Urethral Length
OR: Odds Ratio
RALP: Robotic Assisted Laparoscopic Prostatectomy
SA-PCCOC: South Australian Prostate Cancer Clinical Outcomes Collaborative
